# Dynamic Response-Based Safety Monitoring and Damage Identification of Concrete Arch Dams via PSO–LSTM

**DOI:** 10.3390/s26041136

**Published:** 2026-02-10

**Authors:** Jianchun Qiu, Wenqin He, Changlin Long, Yang Zhang, Xinyang Liu, Pengcheng Xu, Linsong Sun, Changsheng Zhang, Lin Cheng, Weigang Lu

**Affiliations:** 1 College of Hydraulic Science and Engineering, Yangzhou University, Yangzhou 225009, China; mz120231134@stu.yzu.edu.cn (W.H.); 007975@yzu.edu.cn (X.L.); m18994113495@163.com (P.X.); 2 Zhejiang Guangchuan Engineering Consulting Co., Ltd., Hangzhou 310020, China; mz120221147@stu.yzu.edu.cn; 3 Yancheng Water Conservancy Survey, Design and Research Institute Co., Ltd., Yancheng 224000, China; zhangyangycsy@163.com; 4 Jiangsu Hydraulic Engineering Construction Co., Ltd., Yangzhou 225002, China; zhengzhu33@gmail.com; 5 State Key Laboratory of Eco-Hydraulics in Northwest Arid Region, Xi’an University of Technology, Xi’an 710048, China; chenglin@xaut.edu.cn

**Keywords:** dynamic response, damage identification, PSO-LSTM, concrete arch dam, safety monitoring

## Abstract

The measured dynamic response of concrete arch dams under seismic excitation is a typical time series that contains rich information about structural conditions. Safety monitoring based on dynamic responses of arch dam structures is highly important for the timely detection of structural damage and ensuring dam safety. In this study, a PSO-LSTM-based model for safety monitoring and damage identification of arch dam structures was proposed. The method was centered on the long short-term memory (LSTM) neural network, and key hyperparameters were adaptively tuned by the particle swarm optimization (PSO) algorithm to improve monitoring accuracy for nonlinear and nonstationary structural dynamic responses. Structural damage was identified through residual analysis combined with the 3*σ* anomaly detection criterion. Numerical simulations and shaking table model test cases of an arch dam were introduced for validation. The proposed method was compared with the standalone LSTM model and the SSA-LSTM model in terms of the root mean square error (RMSE), mean absolute error (MAE), coefficient of determination (R^2^), and damage identification accuracy. The results showed that the proposed PSO-LSTM method achieved greater accuracy in monitoring the safety of arch dam dynamic responses and effectively identified structural damage, thereby verifying its effectiveness.

## 1. Introduction

As a vital type of large-scale water-retaining structure in hydraulic engineering, arch dams are widely employed in high-head gorge river interception and regulation projects owing to their superior mechanical performance and cost effectiveness, playing an irreplaceable role in flood control, irrigation, water supply, and power generation [1]. However, under extreme dynamic loads such as strong earthquakes, arch dam structures still face potential risks of cracking, spalling, and even overall instability and failure. On the one hand, the complex fluid–structure interaction among reservoir water, sediment layers, the dam body, and the foundation can significantly amplify the seismic response [2,3]; on the other hand, seismic loads impose intense alternating tensile–compressive stresses on the central region of the dam crest, whereas stress concentration often renders the dam–foundation junction the weakest point most prone to severe damage, thereby posing a serious threat to the safety of life and property in downstream areas [4,5].

For post-earthquake safety assessment and structural damage identification of concrete arch dams, traditional damage state evaluation primarily relies on the ratio between the design and measured responses, static monitoring data, and manual inspections [6]. However, these approaches are often insensitive and lack timeliness with respect to local damage, particularly microcracks and internal deterioration in submerged or concealed regions; thus, they are insufficient to support early warning and precise diagnosis. In contrast, structural dynamic responses contain rich information that reflects both global stiffness degradation and local damage evolution, and they are highly sensitive to structural performance deterioration [7,8]. As a result, dynamic responses have become a core data source in modern structural health monitoring (SHM) systems.

To date, multiple technical approaches for dynamic response-based damage identification have been developed. Conventional methods include (1) frequency-domain methods based on modal parameter changes, such as shifts in natural frequencies [9], the modal assurance criterion (MAC), flexibility matrix variations, and curvature mode shape changes [10], and (2) physics-based finite element model updating techniques, in which model parameters are adjusted so that simulated responses match measured data to inversely determine damage location and extent [11,12]. In addition, time-domain methods (e.g., ARMA models, cointegration analysis, and time-series residual analysis), time–frequency analysis methods (e.g., wavelet transform and Hilbert–Huang transform), and energy- or entropy-based feature extraction techniques (e.g., wavelet packet energy entropy, sample entropy, and permutation entropy) are also widely used to capture localized anomalies in nonstationary responses. However, the aforementioned traditional methods generally rely on linear assumptions, modal identifiability, or high-fidelity physical models, and under the highly nonlinear, nonstationary, and multifield coupled conditions induced by strong earthquakes, they often fail to characterize the evolution of minor local damage effectively. Moreover, their generalizability is limited, making them inadequate for meeting the requirements of high accuracy and robustness for real-time safety monitoring of concrete arch dams [13].

In recent years, with advances in sensor technology and computational power, data-driven intelligent damage identification methods have become a research hotspot [14,15]. These methods do not depend on precise physical models but instead learn damage-sensitive features directly from raw or preprocessed dynamic response signals. Techniques such as support vector machine (SVM), deep neural network (DNN), convolutional neural networks (CNNs), and recurrent neural networks (RNNs) have demonstrated superior adaptability and identification accuracy under complex nonlinear and nonstationary conditions. Recent studies have further advanced this field by developing multimodal fusion frameworks that integrate multisensor data and heterogeneous signal representations [16], proposing balanced semisupervised GANs to address the critical challenges of limited labelled data and severe class imbalance in real-world SHM scenarios [17], and introducing novel multiscale feature fusion architectures for uncertainty quantification in laminated structures [18]. Moreover, specialized deep learning models have been tailored for specific sensing modalities, such as the use of 2D CNNs for damage quantification from electromechanical impedance signals [19] and the leveraging of corrosion-induced degradation data to predict bond strength loss in reinforced concrete under acidic environments [20]. These works collectively demonstrate the versatility and increasing sophistication of deep learning approaches in tackling diverse challenges across civil infrastructure health monitoring [21]. Among them, long short-term memory (LSTM) neural networks, as advanced forms of RNNs, incorporate gating mechanisms (input gates, forget gates, and output gates) to effectively capture long-term dependencies and dynamic evolutionary features within time series. It is particularly well-suited for modelling the nonstationary dynamic responses of arch dams subjected to multiple time-varying loads, such as seismic activity, temperature variations, and reservoir water level fluctuations. Owing to its strong capability in damage state classification, trend prediction, and anomaly detection, LSTM has demonstrated considerable potential and significant value for structural health monitoring applications [22].

With the widespread use of high-precision seismographs, strong-motion records from dams now exhibit high signal-to-noise ratios (SNRs), preserving critical dynamic features while minimizing noise interference [23,24]. Moreover, LSTM inherently possesses noise resilience through its gating mechanism, which can suppress random disturbances during training [25]. Given these conditions, dedicated denoising preprocessing is unnecessary; thus, this study directly uses raw acceleration time-series to avoid signal distortion and assess the model’s practicality in real-world monitoring [26,27].

Many studies have explored the application of LSTM and its variants in structural damage identification. For example, based on time-domain dynamic responses, Tanmay Das et al. [28] proposed a hybrid structural damage localization and quantification method that combines gated recurrent units (GRUs) and LSTM neural networks. Chencho et al. [29] utilized an LSTM autoencoder together with impulse response functions (IRFs) for structural damage quantification, accurately identifying and assessing structural damage under conditions of measurement noise and stiffness parameter uncertainty in both single-element and multiple-element damage scenarios. Do-Eun Choe et al. [30] employed LSTM and GRU neural networks for structural damage detection in blades of floating offshore wind turbines (FOWTs). Damage identification techniques based on dynamic responses are nondestructive and can be implemented during the normal operation of structures, enabling continuous structural health monitoring. Moreover, they provide a comprehensive reflection of the global structural behavior, making them particularly suitable for large-scale, highly integrated engineering structures such as arch dams.

Despite its promise, applying LSTM to arch dam damage identification faces challenges. First, model performance is highly sensitive to hyperparameters (e.g., hidden units, learning rate, and time steps). Manual tuning is inefficient and prone to local optima [31,32]. Second, residual noise or sensor anomalies may mislead training and cause overfitting [33,34]. To address these issues, metaheuristic optimization is adopted herein to adaptively tune the LSTM hyperparameters.

To overcome these limitations, metaheuristic optimization algorithms were adopted in this study to adaptively optimize the parameters of the LSTM model, with the aim of improving its stability and identification accuracy under complex engineering conditions. Specifically, particle swarm optimization (PSO) and the SSA were employed to perform global optimization of key LSTM hyperparameters. However, the SSA was found to suffer from rapid loss of population diversity and a tendency toward premature convergence, resulting in poor stability when searching for optimal hyperparameters in high-dimensional continuous spaces.

In contrast, PSO was considered more suitable for adaptive tuning of LSTM hyperparameters because of its simple structure, well-established convergence theory, and efficient exploration capability for continuous variables. Global search in PSO was achieved through the cooperative guidance of individual and population historical best information, and it demonstrated good convergence speed and stability in high-dimensional continuous spaces. Moreover, PSO is characterized by low computational complexity and a small number of control parameters (primarily inertia weights and learning factors), making it easier to integrate with the LSTM model for efficient automatic hyperparameter optimization. These advantages render PSO particularly well-suited for damage identification tasks involving concrete arch dams, which exhibit high dimensionality, strong nonlinearity, and significant noise interference.

Accordingly, to increase the prediction stability and damage identification accuracy of the LSTM model under non-stationary loading conditions, avoid premature convergence and human-induced errors, and improve robustness against noise interference, an optimization framework was constructed via the PSO algorithm with the objective of maximizing classification accuracy or minimizing the loss function. The optimal combination of the hidden layer architecture and learning parameters was automatically searched, and a PSO-LSTM damage identification model was established. To verify the effectiveness of the proposed method for safety monitoring of concrete arch dams, validation studies based on dynamic responses were conducted, including a numerical simulation case of a concrete arch dam and shaking table tests on a physically scaled model. In addition, a standalone LSTM model and an SSA-LSTM model were also developed for baseline comparisons. The performance of the PSO-LSTM model was comprehensively evaluated via three metrics—root mean square error (RMSE), mean absolute error (MAE), and coefficient of determination (R^2^)—to assess the error magnitude, stability, and goodness of fit of the error from multiple perspectives.

The main contributions of this paper are summarized as follows:

(1) A PSO-LSTM intelligent method based on dynamic responses was proposed for safety monitoring and damage identification of concrete arch dam structures under seismic loading.
(2) An optimization framework was established with the objective of maximizing classification accuracy or minimizing the loss function, and the PSO algorithm was employed to adaptively optimize critical hyperparameters of the LSTM network, including the number of hidden-layer neurons, initial learning rate, and maximum training epochs.
(3) A damage detection mechanism based on residuals and the 3σ criterion was proposed, and the effectiveness of the method was validated through both numerical simulation and physical model testing.

The remainder of this paper is organized as follows: Section 2 details the architecture and design principles of the PSO-LSTM damage identification method, along with the formulation of damage indicators and damage decision criteria. Section 3 provides a comprehensive validation of the proposed framework via finite element simulation data and scaled shaking table test results from a concrete arch dam. Finally, Section 4 concludes the study and outlines directions for future research.

## 2. LSTM-Based Damage Identification Method Using Dynamic Responses

### 2.1. Long Short-Term Memory (LSTM) Neural Network

The LSTM neural network is a special type of recurrent neural network (RNN) that is capable of processing and retaining information from long sequences [35,36]. By introducing memory cells and gating mechanisms, LSTM effectively addresses the problems of gradient vanishing and gradient explosion that are commonly encountered by traditional RNNs when dealing with long sequences. An LSTM network consists of an input layer, a hidden layer, a recurrent layer, and an output layer. Each LSTM unit in the hidden layer is composed of three gate mechanisms: the input gate, the forget gate, and the output gate [37].

The forget gate in the LSTM unit determines which information should be discarded from the cell state. It computes a value between 0 and 1 via a sigmoid activation function, indicating the degree to which past information is retained. The calculation is expressed as follows:

(1) $$f_{t}=\sigma \left(W_{f}\cdot \left[h_{t-1},x_{t}\right]+b_{f}\right)$$

where $f_{t}$ denotes the forget gate; $h_{t-1}$ represents the previous output; $x_{t}$ represents the input to the current cell; $W_{f}$ represents the weight matrix of the forget gate; σ represents the sigmoid activation function; and $b_{f}$ represents the bias term.

The input gate determines which new information should be added to the cell state. It consists of two components: a sigmoid layer and a *tanh* layer. The corresponding computation is given by Equation (2). By combining the outputs of the forget gate and the input gate, the cell state is updated, as expressed in Equation (3):

(2) $$\left\{\begin{array}{l} i_{t}=\sigma \left(W_{i}\cdot \left[h_{t-1},x_{t}\right]+b_{i}\right)\\ {\tilde{C}}_{t}=tanh\left(W_{C}\cdot \left[h_{t-1},x_{t}\right]+b_{C}\right) \end{array}\right.$$

(3) $$C_{t}=f_{t}C_{t-1}+i_{t}{\tilde{C}}_{t}$$

where $i_{t}$ denotes the input gate; σ represents the sigmoid activation function; ${\tilde{C}}_{t}$ represents the cell state; *tanh* represents the hyperbolic tangent activation function; $W_{i}$ and $W_{C}$ are the weight matrices associated with the input gate; $b_{i}$ and $b_{C}$ are the bias terms; and the ∗ symbol denotes elementwise multiplication.

The output gate in the LSTM unit is used to determine the information to be output. The computation is formulated as follows:

(4) $$\left\{\begin{array}{l} o_{t}=\sigma (W_{o}\cdot \left[h_{t-1},x_{t}\right]+b_{o})\\ h_{t}=o_{t}tanh(C_{t}) \end{array}\right.$$

where *O_t_* denotes the output gate; *h_t_* represents the output value of the current cell; *W_o_* is the weight matrix of the output gate; and *b_o_* is the bias term.

### 2.2. Hyperparameter Optimization of LSTM Neural Networks

The selection of hyperparameters in LSTM neural networks is crucial for model performance. By appropriately choosing hyperparameters such as the learning rate, the number of LSTM units in the hidden layer, the maximum number of training epochs, and the regularization coefficient, the model’s performance in processing dynamic response signals can be significantly improved. However, these hyperparameters typically need to be set manually. As key factors influencing training efficiency and prediction accuracy, hyperparameters play a decisive role in model effectiveness.

To avoid the empiricism and randomness associated with manual hyperparameter selection, a metaheuristic algorithm was employed in this study to optimize the hyperparameters of the LSTM network. However, not all metaheuristic algorithms were found to be equally suitable for this task. Taking the SSA as an example, although it mimics the foraging behavior of sparrows and exhibits excellent performance on certain low-dimensional benchmark functions, it is prone to rapid loss of population diversity in high-dimensional continuous search spaces—such as those formed by combinations of LSTM hyperparameters including the number of hidden neurons, learning rate, and time-step length—due to an imbalance in its “discoverer–joiner” mechanism. This often leads the algorithm to converge prematurely to local optima. In particular, when applied to dynamic response signals characterized by multiband frequency content and strong transient features, the LSTM architecture optimized by the SSA was susceptible to overfitting noise patterns present in the training data, which could result in either false alarms or missed detections at critical damage instants.

In contrast, the PSO achieves a global search through the cooperative guidance of each particle’s personal best (pbest) and the swarm’s global best (gbest). This mechanism effectively balances exploration and exploitation while demonstrating superior stability in high-dimensional continuous spaces. Moreover, PSO involves fewer control parameters—primarily inertia weights and cognitive/social coefficients—thereby reducing the tuning complexity and allowing the optimization process to focus more effectively on enhancing the LSTM model’s ability to capture nonstationary, abrupt dynamic responses. Consequently, the PSO-LSTM framework not only mitigates overfitting but also accurately identifies transient features in dynamic responses caused by local cracking or stiffness degradation.

Accordingly, the PSO was employed in this study to automatically optimize the hyperparameters of the LSTM neural network. The SSA was additionally implemented as a comparative counterpart to validate the superior prediction stability and damage identification capability of the PSO-LSTM model under nonstationary loading conditions.

#### 2.2.1. PSO Method

**Figure 1:**
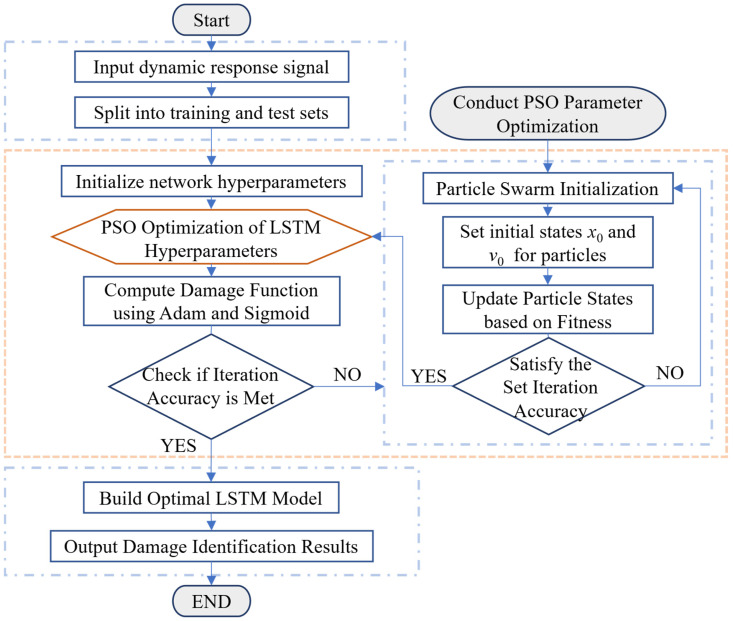
Overall flowchart of the PSO-LSTM model.

Figure 1 illustrates the overall workflow of the PSO-LSTM damage identification model. The PSO algorithm is a swarm intelligence method that simulates the foraging behavior of birds, abstracting each bird as a particle with position and velocity attributes. Particles search for food by sharing information and continuously adjusting their positions and velocities on the basis of their own and neighboring particles’ influences. The fitness of the current position is evaluated to determine its quality, and the process iterates and updates until the optimal solution is found [38,39]. The velocity and position update formulas for the particles are as follows:

(5) $$v_{i}\left(t+1\right)=\omega v_{i}\left(t\right)+c_{1}r_{1}\left(X_{i}^{p}\left(t\right)-x_{i}\left(t\right)\right)+c_{2}r_{2}\left(X_{i}^{g}\left(t\right)-x_{i}\left(t\right)\right)$$

(6) $$x_{i}\left(t+1\right)=x_{i}\left(t\right)+v_{i}\left(t+1\right)$$

where *i* denotes an individual particle, where i=1,2,…,N and *N* are the total number of particles; *t* represents the current time step, and *t* + 1 represents the next time step; *v_i_* is the current velocity of particle *i*, and *x_i_* is its current position; ω is the inertia weight, indicating the particle’s ability to retain its previous velocity; and *c*_1_ and *c*_2_ are learning factors representing the learning rates for convergence to the local best solution (*c*_1_) and the global best solution (*c*_2_), respectively. Their values typically range from 0 to 4, with common settings being *c*_1_ = *c*_2_ = 2; *r*_1_ and *r*_2_ are random numbers uniformly distributed in the range [0, 1], used to introduce randomness into the optimization process; $X_{i}^{p}\left(t\right)$ is the personal best position (local best solution) that particle *i* has achieved thus far; $X_{i}^{g}\left(t\right)$ is the global best position (global best solution) achieved by any particle in the swarm.

#### 2.2.2. SSA Optimization Method

The basic idea of the SSA is inspired by the foraging behavior of sparrows. In the population, each sparrow can adopt one of three behavioral strategies: discoverer (searching for food), joiner (following the discoverer to find food), or scout (monitoring the environment for threats).

**Figure 2:**
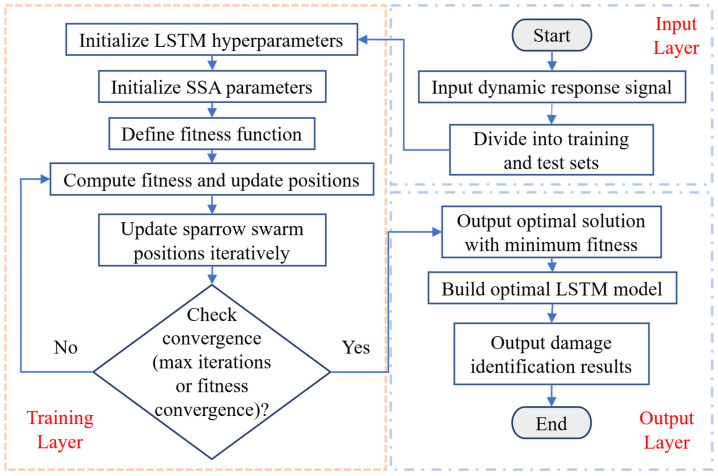
Overall flowchart of the SSA-LSTM model.

The discoverer takes the lead in searching for food sources and emits signals upon discovering food to attract followers and share resources. The joiner locates food by following the discoverer. Moreover, the scout observes the surrounding environment and issues early warnings to alert the group of potential predators, enabling the population to avoid danger [40]. Figure 2 illustrates the overall workflow of the SSA-LSTM damage identification model.

The specific implementation steps of the SSA are as follows:

First, a n*d matrix *X* is constructed to represent the sparrow population:

(7) $$X=\left[\begin{array}{ccccc} x_{1,1} & x_{1,2} & x_{1,3} & \ldots & x_{1,d}\\ x_{2,1} & x_{2,2} & x_{2,3} & \ldots & x_{2,d}\\ x_{3,1} & x_{3,2} & x_{3,3} & \ldots & x_{3,d}\\ \vdots & \vdots & \vdots & \vdots & \vdots \\ x_{n,1} & x_{n,2} & x_{n,3} & \ldots & x_{n,d} \end{array}\right]$$

where *n* represents the number of sparrows in the population and *d* denotes the dimensionality of the optimization problem.

The fitness values *F_X_* of all sparrows for different optimization problems are expressed as follows:

(8) $$F_{X}=\left[\begin{array}{c} f\left(\left[\begin{array}{ccccc} x_{1,1} & x_{1,2} & x_{1,3} & \ldots & x_{1,d} \end{array}\right]\right)\\ f\left(\left[\begin{array}{ccccc} x_{2,1} & x_{2,2} & x_{2,3} & \ldots & x_{2,d} \end{array}\right]\right)\\ f\left(\left[\begin{array}{ccccc} x_{3,1} & x_{3,2} & x_{3,3} & \ldots & x_{3,d} \end{array}\right]\right)\\ \vdots \\ f\left(\left[\begin{array}{ccccc} x_{n,1} & x_{n,2} & x_{n,3} & \ldots & x_{n,d} \end{array}\right]\right) \end{array}\right]$$

Therefore, the position of the discoverer $X_{i,j}^{t+1}$ is updated as follows:

(9) $$X_{i,j}^{t+1}=\left\{\begin{array}{l} X_{i,j}^{t}\cdot \exp\left(-\frac{i}{\alpha \cdot iter_{\max}}\right),R_{2}<ST\\ X_{i,j}^{t}+Q\cdot L,\;R_{2}\geq ST \end{array}\right.$$

where *t* denotes the current iteration number of the sparrow population; j=1,2,3,⋯,d; *iter_max_* is a constant representing the maximum number of iterations; α is a random number uniformly distributed in the range (0, 1]; $R_{2}\left(R_{2}\in \left[0,1\right]\right)$ represents the alarm value; $ST\left(ST\in \left[0.5,1\right]\right)$ denotes the safety threshold; $X_{i,j}$ represents the position of the *i*-th sparrow in the *j*-th dimension; *L* is a 1*d matrix with all the elements equal to 1; and *Q* is a random number following a standard normal distribution.

When $R_{2}<ST$, no predator is present in the surrounding environment. The joiner $X_{i,j}^{t+1}$ updates its position to search for food, following the update formula below:

(10) $$X_{i,j}^{t+1}=\left\{\begin{array}{l} Q\cdot \exp\left(\frac{X_{worst}-X_{i,j}^{t}}{i^{2}}\right),\;i>n/2\\ X_{p}^{t+1}+\left|X_{i,j}^{t}-X_{P}^{t+1}\right|\cdot A^{+}\cdot L,\begin{array}{c} otherwise \end{array} \end{array}\right.$$

where *X_P_* denotes the best position found by the discoverer thus far; *X_worst_* represents the worst position in the current population; *A* is a 1*d matrix with elements randomly assigned as either 1 or −1, satisfying $A^{+}=A^{T}{\left(AA^{T}\right)}^{-1}$; and *L* is a 1*d matrix with all the elements equal to 1.

When i>n/2, the *i*-th joiner has not obtained food and needs to search in other regions. In the sparrow population, approximately 10~20% of sparrows act as scouts, perceiving threats and rapidly migrating to safer areas. The position $X_{i,j}^{t+1}$ of these scout individuals is updated according to the following mathematical expression:

(11) $$X_{i,j}^{t+1}=\left\{\begin{array}{l} X_{best}^{t}+\beta \cdot \left|X_{i,j}^{t}-X_{best}^{t}\right|,\;f_{i}>f_{g}\\ X_{i,j}^{t}+K\cdot \left(\frac{\left|X_{i,j}^{t}-X_{worst}\right|}{\left(f_{i}-f_{w}\right)+\varepsilon }\right),f_{i}=f_{g} \end{array}\right.$$

where *X_best_* denotes the best position in the current search area; *β* is a random number within the range [−1, 1]; *f_i_* represents the fitness value of the current sparrow individual; *f_g_* is the global best fitness value in the entire population; *f_w_* is the worst fitness value in the entire population; and *ε* is a small constant used to prevent division by zero.

When *f_i_* > *f_g_*, the sparrow is located in a marginal area of the population and is at risk of predator attack. When *f_i_* = *f_g_*, even sparrows at the center of the population have encountered danger, and in such cases, they need to move closer to other sparrows to reduce the risk of predation.

### 2.3. Damage Index Development and Damage Assessment

In this study, damage was defined as the irreversible deterioration of structural materials or geometric continuity in concrete arch dams under extreme loading conditions, such as strong seismic excitations. This deterioration was characterized by the initiation and propagation of micro-cracks, localized spalling or crushing of the concrete, unexpected deformations including joint opening/closing and sliding, and a consequent reduction in the global or local stiffness. These physical changes significantly altered the dynamic characteristics of the structure (e.g., natural frequencies, damping ratios, and mode shapes) and were reflected in the seismic response signals, thereby providing a physical basis for vibration-based damage identification.

Under seismic loading, structural damage causes alterations in dynamic characteristics, resulting in nonlinear, abrupt changes in system behavior. These changes are reflected in acceleration, displacement, and other measured response signals through phenomena such as frequency drift, abnormal energy distribution, or waveform distortion. In this study, a damage identification model was developed using an LSTM neural network. First, the network was trained on monitoring data collected under healthy conditions to learn the normal dynamic response patterns of the structure. Subsequently, anomaly detection was achieved by comparing the residuals between the LSTM-predicted responses and the actual measured responses. When the prediction residuals increased significantly and persisted over time, the structural response deviated from the baseline of the healthy state, which could be interpreted as the occurrence of damage, thus enabling high-precision damage identification. The overall procedure is as follows:

(1) Healthy baseline model: An LSTM-based baseline model was trained using measurement data acquired from the undamaged structure, enabling the prediction of dynamic responses ${\hat{y}}_{t}$ under normal operating conditions.
(2) Residual sequence: The residual *e_t_* was calculated as the difference between the measured signal *y_t_* and the predicted value ${\hat{y}}_{t}$, expressed as $e_{t}=\left|y_{t}-{\hat{y}}_{t}\right|$. Structural damage induced significant deviations of *e_t_* from its baseline statistical distribution—specifically, exceeding the threshold defined by the mean ± 3*σ*.

In statistics, the 3*σ* criterion (also known as the 68–95–99.7 rule) is a fundamental property of the normal distribution, characterizing the concentration tendency of data dispersion. In engineering-based damage identification, this principle has been widely adopted in residual analysis to detect significant anomalies caused by structural deterioration. Within the context of structural health monitoring, the residual sequence *e_t_* under the healthy state was assumed to follow a normal distribution with zero mean and constant variance. When structural damage occurs, nonlinear changes in the dynamic response lead to a shift in the statistical properties of the residuals *e_t_*, including deviations in the mean, increased variance, or non-Gaussian distribution patterns, thereby indicating a departure from the established baseline behavior.

If the residual *e_t_* under the healthy state was assumed to follow a normal distribution, i.e., *e_t_* ∼ *N* (0, *σ*^2^), then the normal residual range was defined as −3*σ* ≤ *e_t_* ≤ 3*σ*. Upon damage occurrence, data points exceeding this threshold were observed. For high-safety-critical structures (e.g., nuclear power plants), a more stringent 4*σ* threshold (corresponding to an anomaly probability of 0.006%) was adopted, whereas in environments with higher noise levels, the threshold could be relaxed to 2*σ* (anomaly probability of 4.55%), provided that additional indicators—such as the duration of sustained exceedance of the threshold—were incorporated for comprehensive assessment.

Therefore, when $\left|y_{t}-{\hat{y}}_{t}\right|\geq 3\sigma$, seismic loading was likely to induce structural damage in the concrete dam. In this study, damage occurrence was determined by comparing the absolute residual $\left|y_{t}-{\hat{y}}_{t}\right|$ with the 3*σ* threshold, enabling reliable detection of significant structural deterioration.

## 3. Examples

### 3.1. Numerical Simulation Example

#### 3.1.1. Dynamic Simulation of a Concrete Arch Dam

**Figure 3:**
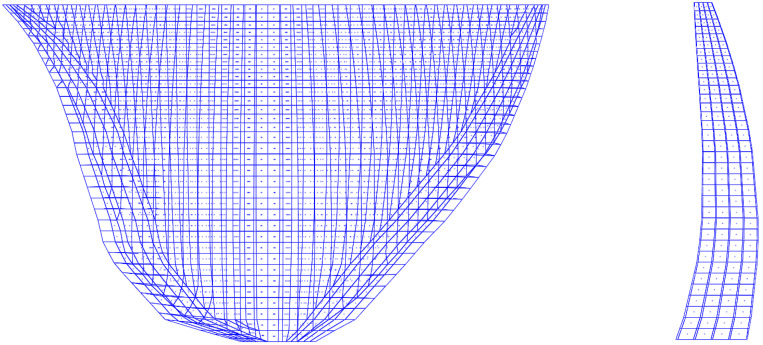
Plan layout of the dam.

A three-dimensional dynamic finite element simulation was conducted for a concrete arch dam as a case study. The normal reservoir water level of the project is 1880 m. As shown in Figure 3, the dam has a crest elevation of 1885.0 m, a lowest foundation elevation of 1580.0 m, and a maximum height of 305.0 m. The crest width is 16.0 m, and the base thickness is 63.0 m.

**Figure 4:**
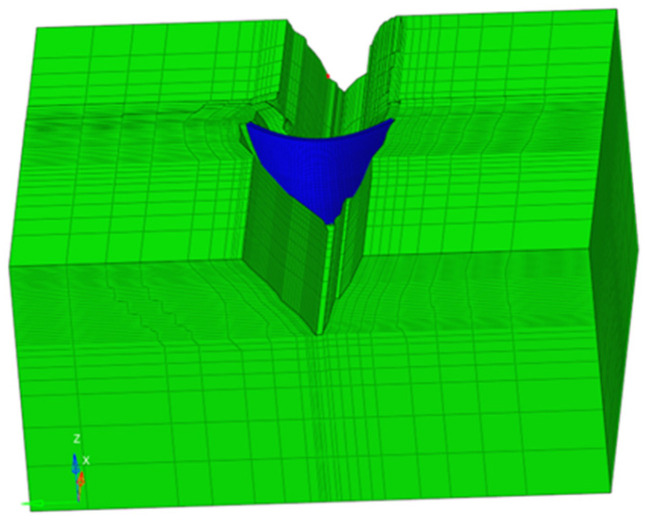
Finite element model of the arch dam.

Figure 4 illustrates the finite element model of this arch dam. The model extends two times the dam height in both the upstream and downstream directions and 1.5 times the dam height in the foundation direction. For simplification, lateral joints were not considered in the numerical simulation. An 8-node hexahedral C3D8R element was used for meshing, resulting in a total of 50,714 elements and 56,921 nodes, with 4696 elements and 6085 nodes specifically for the dam body.

In this simulation, all foundation materials were assumed to be uniform, and the foundation was treated as a massless base. Rayleigh damping was applied to the dam body. To simulate damage in the concrete arch dam, the concrete damage plasticity (CDP) model was adopted as the constitutive model for the dam body, assuming identical failure criteria in all directions. The damage factor obtained through the CDP model visually represents the damage state [41]. The added mass method proposed by Westergaard was adopted to account for the dynamic hydrodynamic pressure. The formula for calculating the added mass according to Westergaard is as follows:

(12) $$m=\frac{7}{8}\rho_{water}\sqrt{H\left(H-Z\right)}$$

where m represents the added water mass; $\rho_{water}$ denotes the density of water; *H* represents the reservoir water depth; and *Z* represents the depth at the location of interest.

**Figure 5:**
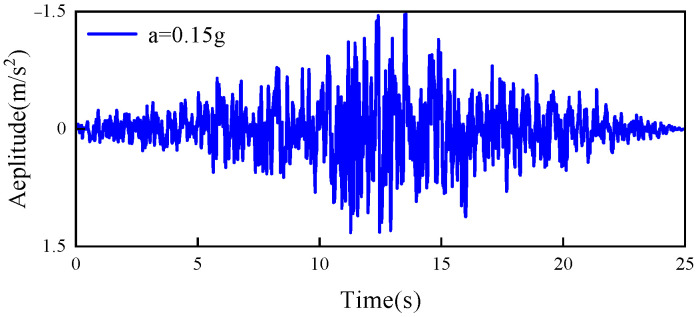
Artificial seismic waves.

To investigate the damage evolution of this concrete arch dam under dynamic loads, an earthquake wave load was designed, as illustrated in Figure 5, with a peak acceleration of a = 0.15 g and a duration of 25 s. The seismic load was applied in the direction along the river flow.

**Figure 6:**
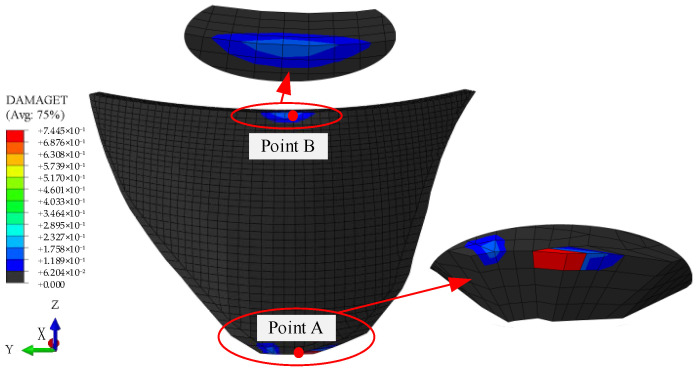
Final damage outcome of the arch dam.

**Figure 7:**
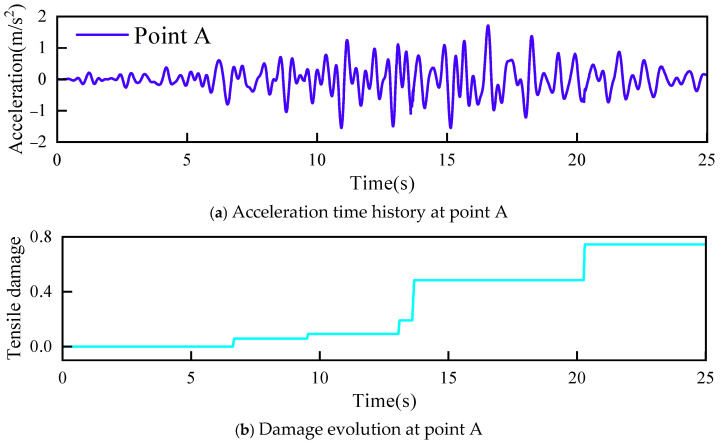
Acceleration time history and damage evolution at point A.

**Figure 8:**
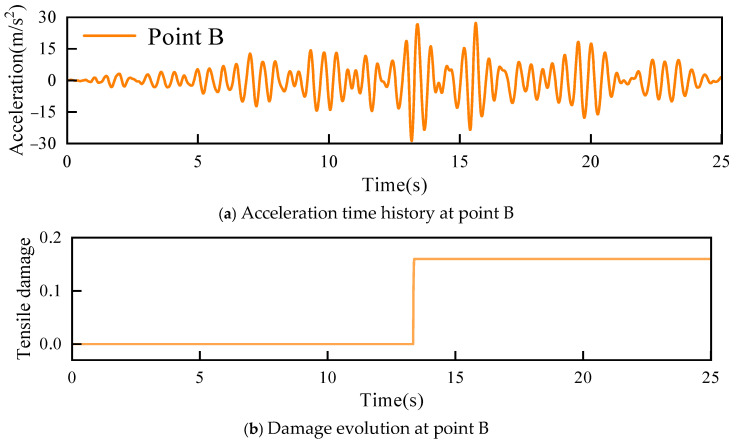
Acceleration time history and damage evolution at point B.

Figure 6 shows the final damage distribution of the dam body. Damage occurred at the crown and heel of the concrete arch dam, and the damage at the heel was more severe. Acceleration responses at point A at the heel and point B at the dam crest were therefore selected for damage analysis. The acceleration response time histories and damage evolution histories at point A and point B are presented in Figure 7 and Figure 8, respectively.

#### 3.1.2. Damage Identification on the Basis of Acceleration Response

To eliminate subjectivity and human-induced bias in hyperparameter selection, the PSO was employed to automatically optimize key hyperparameters of the LSTM neural network, including the number of hidden layer neurons (*hd*), the initial learning rate (*lr*), and the maximum number of training epochs (*E*).

The search spaces were defined as [30, 200], [0.01, 0.2], and [30, 200], respectively. These ranges were determined on the basis of a comprehensive consideration of the theoretical understanding of the LSTM architecture [42], empirical guidelines in deep learning practice [43,44], and computational efficiency constraints.

The range [30, 200] for the number of hidden neurons ensures sufficient model capacity to capture the complex nonlinear dynamics inherent in seismic response signals, while avoiding excessive complexity that could lead to overfitting and increased computational cost. The initial learning rate interval [0.01, 0.2] strikes a balance between training stability and convergence speed, preventing slow convergence due to overly small values or loss function oscillations caused by excessively large rates. The maximum number of epochs is bounded within [30, 200] to provide adequate training iterations, whereas an early stopping mechanism is implemented to halt training once convergence is achieved, thereby avoiding unnecessary computations.

The overall design of the search space follows the principle of combining empirical guidance with practical constraints. It refers to default configurations in mainstream deep learning frameworks and is adaptively adjusted according to the scale of the dataset and the specific requirements of the damage identification task in this study. This well-defined parameter space enables efficient optimization by the PSO algorithm, avoids computationally expensive blind searches, and establishes a rational and feasible boundary for hyperparameter exploration, thus facilitating the construction of a high-precision PSO-LSTM model for structural damage identification.

The particle swarm size was set to 10, the number of iterations was set to 50, and the error tolerance was set to 1 × 10^−5^. The inertia weight was set to vary between *W_min_* = 0.8 and *W_max_* = 1.2, with the cognitive and social learning factors both set to *c*_1_ = *c*_2_ = 2. The particle dimension was set to 3, with initial position *x_0_* and initial velocity *v*_0_. Table 1 provides a detailed list of the optimization parameters, and the optimization results are presented in Table 2.

To validate the good convergence speed and stability of the PSO-LSTM model under complex, nonstationary dynamic response conditions, this study introduced the SSA for optimizing the LSTM model. However, it should be noted that the SSA was utilized here as a comparative algorithm to highlight the advantages of PSO in optimizing LSTM models. When the SSA is used for optimization, several parameters need to be set, including the population size, maximum number of iterations, number of optimized parameters, and lower and upper bounds of the parameter values. In this study, the sparrow population size was set to 10, and the maximum number of iterations was set to 20. Using the SSA algorithm, four hyperparameters of the LSTM model were optimized: the number of hidden layer nodes (*hd*), the maximum number of training epochs (*E*), the initial learning rate (*lr*), and the L2 regularization coefficient. Table 3 provides a detailed list of the optimized parameters.

For the single LSTM neural network model, this study set the number of hidden layer nodes to 50, the initial learning rate to 0.01, the *L*2 regularization coefficient to 0.0001, and the maximum number of training epochs to 100 through manual tuning. All the models were trained based on the acceleration response at point A and used the same input dimension, with a time step length of 50. To prevent overfitting, a dropout rate of 0.2 was uniformly applied. The training data were derived from the first 2 s of the structural response under undamaged conditions, comprising a total of 200 sampling points. The specific parameter values for each model are listed in Table 2.

The optimal parameter combination of the PSO-LSTM model obtained through the PSO algorithm was as follows: number of hidden layer nodes (*hd*) = 83, maximum training epochs (*E*) = 100, and initial learning rate (*lr*) = 0.0541. The optimization results obtained via the SSA algorithm were *hd* = 132, *E* = 149, *lr* = 0.0237, and the *L*2 regularization coefficient = 0.098.

**Figure 9:**
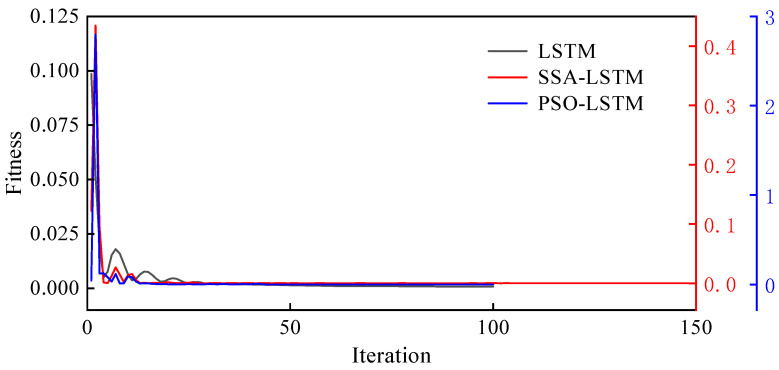
Model convergence curve.

The loss function was used as the fitness value to evaluate model performance. Figure 9 presents the convergence curves of the models during training. As observed in the figure, the PSO algorithm demonstrated superior performance in optimizing the LSTM model compared with the unoptimized model. The optimized model achieved lower loss values on both the training and validation datasets, indicating stronger fitting and generalization capabilities. Moreover, the PSO-optimized LSTM model reached a low loss value within fewer iterations, reflecting higher training efficiency.

Since the dam remained in a stable condition without any structural damage during the first 2 s, the acceleration response at point A over this initial 2 s interval was used as input to train the PSO-LSTM damage identification model. The model was thereby trained to learn the dynamic response characteristics of the undamaged state. The model was subsequently employed to predict and output responses for the time period from 2 to 25 s, enabling damage identification of the arch dam on the basis of its dynamic response signals. Specifically, a concrete damage plasticity (CDP) constitutive model was used in the numerical simulation to generate an acceleration time history of 25 s, with a sampling frequency of 100 Hz, resulting in a total of 2501 time steps. The first 2 s (i.e., 200 data points) were used as the training set, corresponding to the undamaged state of the structure, and were employed to establish a health baseline model. The subsequent 23 s (2301 data points) were used as the test set, which included the damage evolution process, and were utilized for model prediction and damage identification validation. A single split combined with a rolling prediction strategy (without cross-validation) was adopted, aligning with the structural health monitoring (SHM) paradigm of “health baseline modelling followed by online anomaly detection.” The input sequence length was set to 50, meaning that every 50 consecutive time steps were used as input to predict the response at the next time step. The damage identification results produced by the model were then compared with the measured values to evaluate the effectiveness and accuracy of the proposed approach.

**Figure 10:**
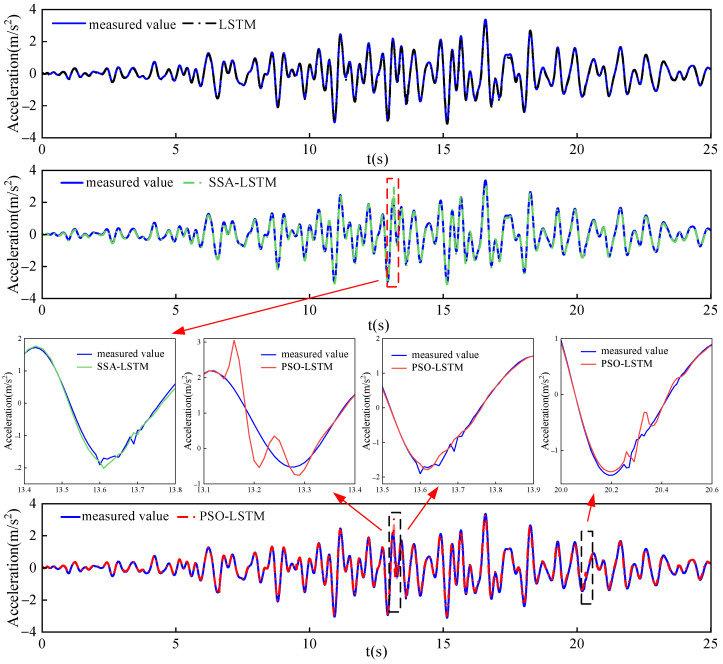
Comparison of model damage detection results with measured values.

Figure 10 presents the damage prediction results of each model for the arch strong earthquake response of the arch dam, where the outputs of the LSTM, SSA-LSTM, and PSO-LSTM models correspond to the predictions of their respective models.

The output of the standalone LSTM model is generally smooth and broadly follows the trend of the measured value; however, it fails to effectively reproduce the transient, abrupt features present in the measured value, indicating insufficient sensitivity to nonstationary responses induced by localized damage. In contrast, the SSA-LSTM model exhibits a pronounced anomalous fluctuation at approximately 13.6 s and displays relatively high volatility during other intervals. This behavior likely stems from the tendency of the SSA algorithm to become trapped in local optima when operating in high-dimensional continuous search spaces, leading to suboptimal hyperparameter configurations for the LSTM network and consequently degrading its generalization capability and stability.

By comparison, the PSO-LSTM model was able to capture the significant dynamic variations present in the measured value accurately. Its prediction exhibited clear deviations consistent with actual damage-induced anomalies at approximately 13–14 and near 20 s, and the predicted waveform was found to closely align with the measured signal. This demonstrated that the hyperparameter configuration obtained through PSO effectively enhanced the model’s capacity to extract damage-sensitive features. Overall, the PSO-LSTM model not only maintains consistency with the measured response in terms of global trends but also exhibits superior fidelity in reconstructing fine-grained details during damage-sensitive periods. These results validate the effectiveness of the proposed optimization strategy in improving both the robustness and damage identification accuracy of the model.

To enhance the clarity of the results, the residual $e_{t}=\left|y_{t}-{\hat{y}}_{t}\right|$ between the measured signal $y_{t}$ and the predicted value ${\hat{y}}_{t}$, along with the corresponding 3σ threshold, was computed. The 3σ values for the standalone LSTM model, SSA-LSTM model, and PSO-LSTM model were 0.3, 0.25, and 0.22, respectively. Structural damage in the concrete arch dam caused by seismic loading was considered to have occurred when $\left|y_{t}-{\hat{y}}_{t}\right|\geq 3\sigma$.

**Figure 11:**
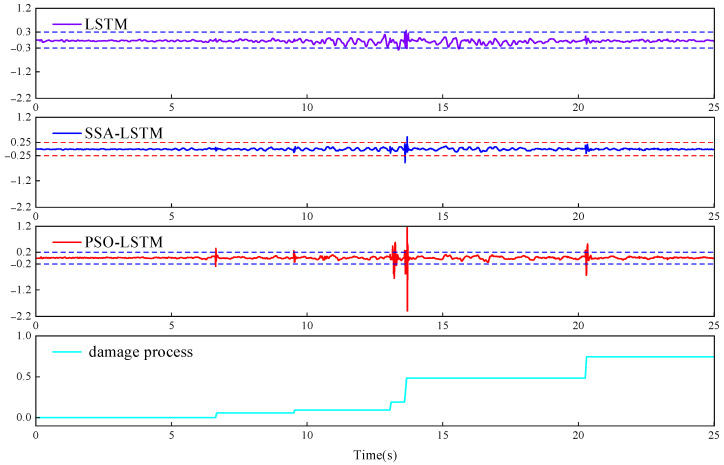
Residual comparison and damage evolution history at point A.

Figure 11 presents the residual comparison and the damage evolution history at point A. To facilitate visual comparison, the first three subplots in Figure 11 are drawn using the same vertical axis scale. The PSO-LSTM model demonstrated the best performance in damage detection, with the highest accuracy and most distinct response. The residuals showed abrupt changes at 6.63 s, 9.52 s, 13.18 s, 13.71 s, and 20.33 s, all exceeding the 3σ limit, and these instants fully matched the actual damage progression at point A, indicating structural failure. The SSA-LSTM model exhibited moderate detection accuracy and a relatively clear response, with a noticeable residual spike only at 13.69 s, surpassing the 3σ threshold and indicating damage occurrence. In contrast, the standalone LSTM model showed the poorest performance in damage detection; its prediction error was relatively large, and significant deviations were present at critical time points, revealing weak adaptability to changes in structural damage.

The residual comparison further revealed differences in damage recognition capabilities among the models at critical moments. Owing to the lack of an adaptive mechanism for hyperparameters, the fixed structure of the LSTM model struggled to balance fitting overall trends and was sensitive to local abrupt changes, leading to ineffective responses at several key nodes, such as 6.63 s and 13.18 s, with noticeable prediction errors. Although the SSA-LSTM model introduces an optimization mechanism, the SSA tends to prematurely converge in high-dimensional spaces, resulting in suboptimal hyperparameter combinations that do not fully exploit the sequential modelling potential of LSTM. This model captured only one damage event near 13.69 s and was less responsive during other periods. On the other hand, the PSO-LSTM model benefits from the efficient global search capability of the PSO algorithm in hyperparameter space, successfully configuring a network structure that combines high sensitivity with strong robustness. The residuals significantly changed at all five known damage moments, closely matching the actual damage progression at point A. This finding verified the superiority of the method in the simultaneous identification of multistage damage.

**Figure 12:**
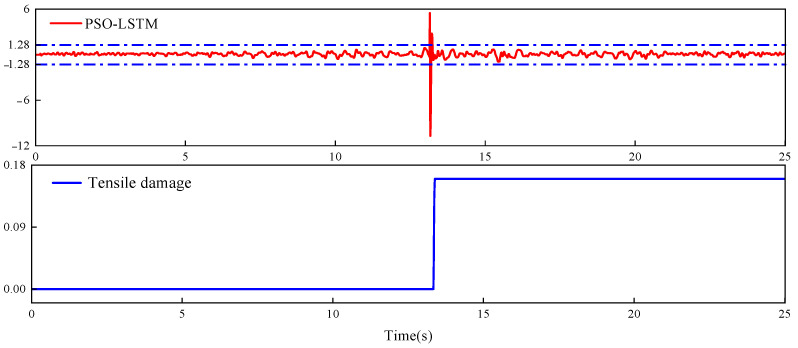
Residual results and damage evolution history at point B.

The PSO-LSTM damage identification model was used to analyse damage at point B. Figure 12 shows the residual values and damage evolution history at point B. A sharp peak in the residual was observed at approximately 13.22 s, while the residuals remained within the range of 3σ = 1.28 at all other times. This indicated that the PSO-LSTM model successfully identified the occurrence of damage at point B at 13.22 s, which was consistent with the fluctuation pattern of the actual damage progression.

In general, evaluating model performance based on a single metric alone is often insufficient to fully capture its behavior in complex engineering scenarios. This paper adopted three evaluation metrics, the root mean square error (RMSE), the mean absolute error (MAE), and the coefficient of determination (R^2^), to comprehensively assess the predictive performance of the integrated safety monitoring and damage identification model for concrete arch dam dynamic responses from multiple perspectives, including error magnitude, stability, and goodness of fit.

Further analysis of the data in Table 4 revealed that the PSO-LSTM model exhibited the best predictive performance, with RMSE, MAE, and R^2^ values of 0.0674, 0.0318, and 0.9981, respectively. The RMSE and MAE values were significantly lower than those of the other models, indicating stricter control overprediction deviations during the reconstruction of strong-motion responses and enabling high-fidelity recovery of the measured acceleration time histories. Moreover, the R^2^ value, which was close to 1, demonstrated an extremely strong correlation between the model output and the measured signals, reflecting excellent dynamic tracking capability. By comparison, the SSA-LSTM model achieves RMSE, MAE, and R^2^ values of 0.0742, 0.0529, and 0.9897, respectively. Although these results were better than those of the basic LSTM model, a noticeable gap remained in terms of error suppression and fitting accuracy. The standalone LSTM model yielded the worst performance across all the metrics, which indicated that, without hyperparameter optimization, it was unable to adequately adapt to the nonlinear and nonstationary characteristics of the arch dam’s dynamic responses.

In summary, the PSO-LSTM model significantly outperforms the other models across multiple prediction performance metrics. This not only validated its effectiveness as a high-precision tool for dynamic response monitoring but also demonstrated its superior ability to extract damage-sensitive features and identify anomalous events. Notably, within the framework of this study, accurate prediction of dynamic responses was a prerequisite for reliable damage identification: only when a model could faithfully reproduce the true structural behavior under both normal and abnormal conditions could residual errors or prediction deviations effectively reveal potential damage. Therefore, the high prediction accuracy of the PSO-LSTM model implies that it could provide timely and trustworthy early warning information in practical engineering applications. This enables engineers to detect structural anomalies at the earliest possible stage and implement timely interventions, thereby substantially enhancing the safety assurance level of concrete arch dams throughout their entire service life.

### 3.2. Dynamic Model Experimental Example of an Arch Dam

#### 3.2.1. Arch Dam Model and Arranged Sensors

**Figure 13:**
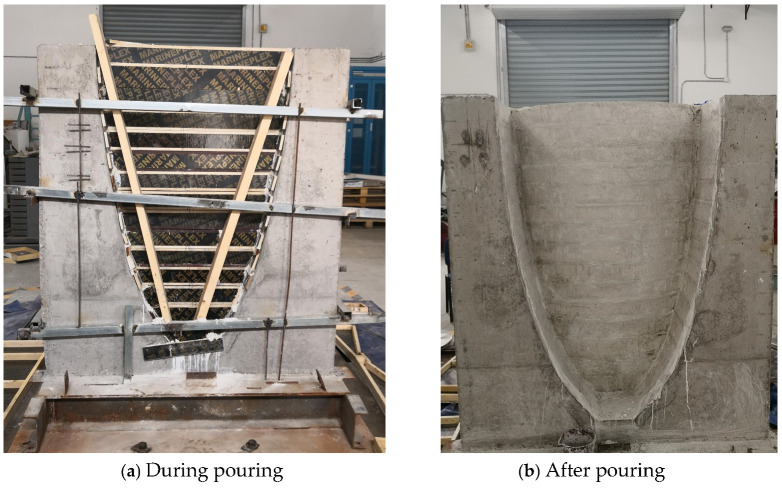
Physical model of the arch dam.

A shaking table model test was selected as a case study to further validate the effectiveness of the proposed method. Figure 13 shows the physical model of the arch dam, which consists of four main components: the dam body, foundation, abutments, and hillside slopes. The dam body had a height of 1.5 m, a maximum crest width of 1.0 m, a crest thickness of 6 cm, and a base thickness of 11 cm. From the top of the dam, the model extended 40 cm downstream, 18 cm upstream, and 14 cm on each side toward the banks. Additionally, part of the foundation was simulated with a thickness of 0.13 m. The dam body, foundation, and abutments were all constructed using simulated concrete material, with a total volume of 0.70 m^3^ and a mass of 2.17 tons [41]. Considering that under dynamic loading, the boundary displacements in actual engineering models are much smaller than those within the dam body, the hillside slopes were modelled using rigid concrete structures to simulate high stiffness. Therefore, the total weight of the entire model structure was 3.86 tons, which fell within the safe load capacity of the shaking table. The shaking table model test has already been described by the authors in previously published literature, and more detailed information can be found in this reference [41].

**Figure 14:**
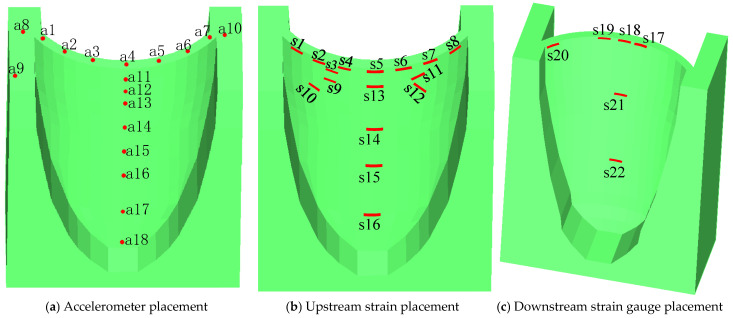
Sensor layout schematics.

**Figure 15:**
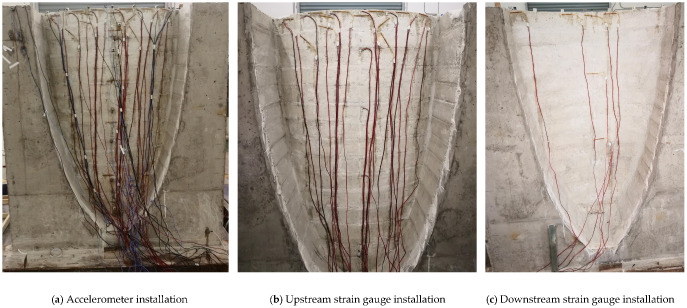
Physical layout of sensors.

The elevation of the dam base was first set as 0.0 m, with the dam crest elevation consequently defined as 1.5 m. The schematic layout of the sensor array is illustrated in Figure 14, whereas the actual installation configuration of the sensors is presented in Figure 15.

In this study, piezoresistive accelerometers and resistance strain gauges were deployed at different locations and elevations of the arch dam structure. By connecting these sensors to a data acquisition system, the dynamic response and strain of the structure were effectively measured. In addition, an accelerometer was placed on the shaking table platform to monitor its acceleration response. The experiment simulated various dynamic loading conditions. This paper focused on the loading case that induced structural damage, for which damage identification analysis of the arch dam’s dynamic response signals was conducted. In this scenario, the structure initially remained undamaged; as the dynamic load progressed, damage began to develop and gradually intensified.

#### 3.2.2. Damage Identification of the Arch Dam Model

**Figure 16:**
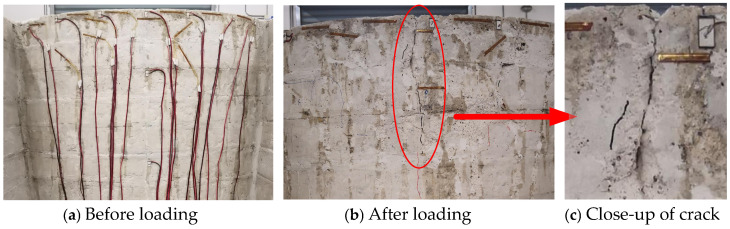
Comparison of the arch crown at the dam crest before and after dynamic loading.

**Figure 17:**
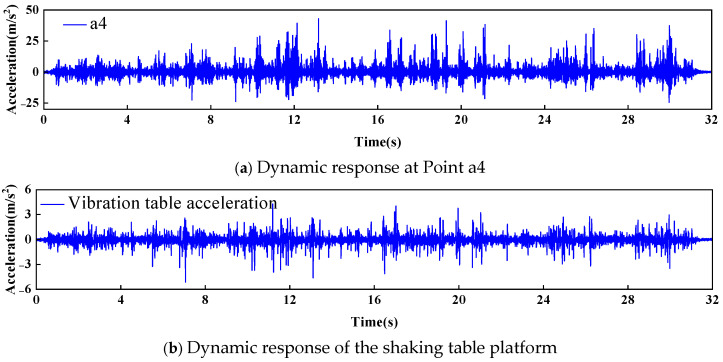
Dynamic response signals of the measurement points in the arch dam model.

During the application of dynamic loading, cracks appeared and gradually expanded only in the middle of the arch crown of the arch dam model, whereas no damage was observed in other areas. Figure 16 shows a comparison of the arch crown at the dam crest before and after dynamic loading, clearly revealing visible cracking after loading. To analyse the dynamic response of the arch dam model under damaged conditions, this section focuses on measurement point a4, which is located at the arch crown of the dam crest. The acceleration signal from the shaking table platform was used as the reference response, which represented the undamaged state. The acceleration responses of all the measurement points are shown in Figure 17.

The acceleration response was input into the model, and the data from the first 2 s corresponding to the undamaged state were used to train the PSO-LSTM model to learn the characteristic response patterns of the healthy structure. To further analyse when damage such as cracking occurred near the crown of the dam crest, three representative strain measurement points, s4, s5, and s6, located in the vicinity of the potential damage zone, were selected for detailed investigation.

**Figure 18:**
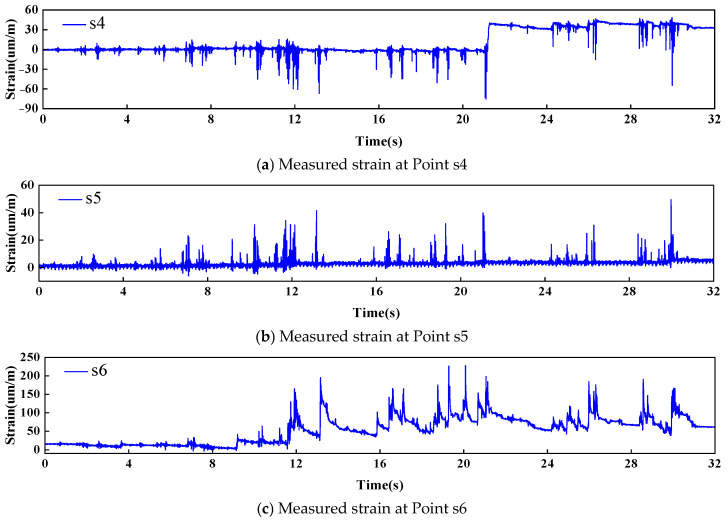
Measured strain responses at each strain gauge.

Figure 18 shows the measured strain responses at these points. As shown in the figure, the initial mean strain values at all the points were zero, indicating linear elastic and recoverable deformation. At t = 5.8s, t = 5.8s, and t = 5.96s, the plastic strain begins to develop at points s4, s5, and s6, respectively, as indicated by a deviation of the mean from zero. Notably, abrupt changes occurred at s4 at t = 21.52s and at s6 at t = 9.46s, with another significant change observed at s6 at t = 13.14s. In contrast, the magnitude and variation in plastic strain at point s5 were relatively small. Therefore, plastic deformation occurred at measurement points s4, s5, and s6.

**Figure 19:**
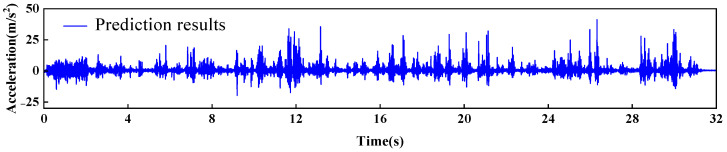
Predicted acceleration response at measurement point a4.

**Figure 20:**
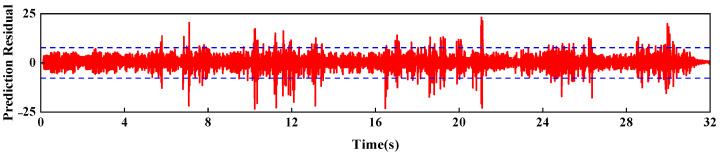
Predicted residuals of the dynamic response at Point a4 (3*σ* = 5.23).

Figure 19 predicts the acceleration response at measurement point a4. Figure 20 shows the prediction residuals of the dynamic response at measurement point a4. As shown in the figure, an anomalous fluctuation in the prediction residuals occurred at t = 5.8 s, indicating the onset of damage in the arch dam structural model. Another anomalous fluctuation appeared between t = 10.21 s and t = 13.40 s, which corresponded to the abrupt change observed in the measured strain at point s6. At t = 21.33 s, the prediction residuals exhibited the largest anomalous fluctuation, which was consistent with the sudden change recorded at strain gauge s4 at t = 21.52 s, suggesting further progression of damage in the arch dam structural model.

The PSO-LSTM damage identification model was applied to detect damage in the arch dam structural model on the basis of dynamic response signals. The model accurately identified the initial damage at t = 5.8 s and captured the subsequent damage progression at t = 10.21 s and t = 21.33 s.

## 4. Conclusions

A method for safety monitoring and damage identification of concrete arch dams on the basis of dynamic responses was proposed in this study, centered on a particle swarm optimization long short-term memory (PSO-LSTM) model. A PSO-LSTM damage identification model was constructed, in which the LSTM neural network was adaptively optimized by the PSO algorithm, enabling high-accuracy damage identification of arch dams via dynamic response signals. The standalone LSTM and SSA-LSTM models were selected for baseline comparisons. The prediction residuals were analysed in conjunction with the 3*σ* anomaly detection criterion, and comprehensive evaluations were conducted across multiple metrics to verify the convergence stability, damage identification accuracy, and robustness of the proposed model under complex operating conditions.

To validate the effectiveness of the proposed method, a case study was conducted on a concrete arch dam. In addition, shaking table test data from a scaled-down arch dam model were employed to experimentally verify the performance of the PSO-LSTM model, further demonstrating the engineering applicability of the proposed approach.

The main research conclusions are as follows:

(1) The PSO algorithm was employed to optimize key hyperparameters of the LSTM network, including the number of neurons in the hidden layer, maximum training epochs, and initial learning rate. This optimization effectively reduced the subjective bias caused by manual parameter tuning and enhanced the accuracy of structural damage identification from dynamic responses. Comparative results demonstrated that the PSO-LSTM model could accurately capture abrupt changes in dynamic responses, resulting in increased sensitivity and reliability. In terms of damage detection accuracy and overall performance, the PSO-LSTM model outperformed both the SSA-LSTM and standalone LSTM models.
(2) The performance of the models was comprehensively evaluated via four quantitative metrics: the root mean square error (RMSE), mean absolute error (MAE), mean absolute percentage error (MAPE), and coefficient of determination (R^2^). The PSO-LSTM model achieved the best performance across all the evaluation metrics, confirming its superiority in damage identification for concrete arch dams on the basis of dynamic response signals. This hybrid model demonstrated a stronger ability to extract discriminative features from complex dynamic signals, effectively capturing the nonstationary behavior of structures. This study provides a robust technical foundation for real-time monitoring and structural safety assessment in practical engineering applications.
(3) To further validate the effectiveness of the PSO-LSTM-based damage identification method, shaking table tests were conducted on a scaled-down arch dam model. The test results revealed that the PSO-LSTM model could accurately identify the onset of structural damage and clearly capture the subsequent damage progression. These identification outcomes were highly consistent with the observed crack initiation and propagation processes during the tests, fully verifying the effectiveness of the proposed model in real-world engineering scenarios.
(4) While the proposed PSO-LSTM-based damage identification framework demonstrates strong performance and practical feasibility, there remains scope for further enhancement and extension. In the present study, the model primarily focused on structural dynamic responses under a single seismic excitation, without explicitly incorporating multisource excitation effects such as dam–reservoir interaction. In addition, the experimental verification was conducted using a scaled arch dam model, which, although effective in validating the fundamental applicability of the method, represents a simplified approximation of real-world engineering conditions. Future research will therefore aim to broaden the applicability of the proposed approach by considering more complex excitation scenarios and structural–environmental coupling effects. Moreover, transfer learning techniques will be introduced to improve the adaptability and generalization capability of the model under varying seismic inputs and operational conditions. Further efforts will also be devoted to extending the method to prototype dams and exploring its feasibility for real-time or online implementation, thereby promoting the practical deployment of the proposed framework in structural health monitoring systems for concrete arch dams.

## Figures and Tables

**Table 1:** Parameter settings of the PSO-LSTM optimization algorithm.

Swarm Size	Maximum Iteration Number	Optimized Parameters	Lower Bound of Optimized Parameters	Upper Bound of Optimized Parameters
10	50	*hd*	[30, 0.01, 30]	[200, 0.2, 200]
		*lr*		
		*E*		

**Table 2:** Model parameter settings.

Model	*hd*	*E*	*lr*	*L*2	Step	Dropout Ratio	Number of Points
LSTM	50	100	0.01	0.0001	50	0.2	200
SSA-LSTM	132	149	0.0237	0.098	50	0.2	200
PSO-LSTM	83	100	0.0541	/	50	0.2	200

**Table 3:** Parameter settings of the SSA-LSTM optimization algorithm.

Population Size	Maximum Iteration Number	Optimized Hyperparameters	Lower Bound of Parameters	Upper Bound of Parameters
10	20	*hd* *E* *lr* *L*2	[30, 30, 0.01, 0.0001]	[200, 200, 0.2, 0.1]

**Table 4:** Evaluation of the damage identification results for each model.

Model	Evaluation Metrics		
	RMSE	MAE	*R* ^2^
LSTM	0.0783	0.0628	0.8577
SSA-LSTM	0.0742	0.0529	0.9897
PSO-LSTM	0.0674	0.0318	0.9981

## Data Availability

The dataset provided in this article is not easily accessible as it is part of ongoing research. Requests to access the dataset should be sent to [qiujc@yzu.edu.cn, mz120231134@stu.yzu.edu.cn].
